# The effectiveness of smart healthcare for patients with rheumatoid arthritis: A systematic review and meta-analysis

**DOI:** 10.1371/journal.pone.0340074

**Published:** 2026-01-08

**Authors:** Yin Yu, Meijiao Wang, Hejing Pan, Lin Huang, Haichang Li, Xuanlin Li

**Affiliations:** School of Basic Medical Sciences of Zhejiang Chinese Medical University, Binjiang District, Hangzhou, China; Prime Hospital LLC, UNITED ARAB EMIRATES

## Abstract

**Objectives:**

This study aimed to evaluate the impact of smart healthcare interventions on disease activity, self-efficacy, self-management, functional levels, and quality of life in rheumatoid arthritis (RA) patients, as well as any associated adverse events.

**Method:**

A systematic search was conducted in PubMed, EMBASE, and Cochrane Library for randomized controlled trials (RCTs) from inception to August 2024, using relevant medical subject headings and keywords. The Cochrane risk of bias tool was employed to assess bias risk. A random effects model was used, calculating the standardized mean difference (SMD) and 95% confidence intervals (CIs). Heterogeneity was assessed with Chi-square and I^2^ tests.

**Result:**

This meta-analysis included 18 RCTs published between 2015 and 2024, with follow-up periods ranging from 3 months to 1 year. Results revealed a significant reduction in Disease Activity Score 28 (DAS28) in the smart healthcare intervention group compared to controls [SMD 95% CI: −0.22 (−0.38, −0.05), I^2^ = 28.8%, P = 0.011]. Additionally, quality of life showed significant improvement as measured by EQ-5D scores [SMD 95% CI: 0.16 (0.01, 0.32), I^2^ = 0.0%, P = 0.033]. Significant improvement was also observed in functional capacity [SMD 95% CI: −0.30 (−0.54, −0.06), I^2^ = 0.0%, P = 0.014]. However, no statistically significant effects were found for self-efficacy [SMD 95% CI: 0.12 (−0.12, 0.35), I^2^ = 0.0%, P = 0.340], self-management ability [SMD 95% CI: 0.11 (−0.17, 0.39), I^2^ = 0.0%, P = 0.451], or RA knowledge [SMD 95% CI: 0.46 (−0.14, 1.07), I^2^ = 75.98%, P = 0.133].

**Conclusion:**

Smart healthcare interventions show promise in reducing disease activity and improving physical function and quality of life in patients with rheumatoid arthritis. While these findings support the potential value of digital health solutions in RA management, further validation through more rigorous methodological designs is needed to confirm long-term effectiveness and clinical utility.

## Introduction

Rheumatoid arthritis (RA) is a prevalent systemic autoimmune disease characterized by symmetrical polyarticular pain, primarily affecting synovial tissues and surrounding soft tissues [1–3]. Its estimated prevalence varies significantly, being higher in industrialized countries, with age-standardized rates of 0.38%, 0.35%, and 0.10% in North America, Western Europe, and Southeast Asia, respectively [2,4,5]. RA disproportionately affects women [6–9], with sex ratios ranging from 4:1 in younger populations to 2:1 in older adults, likely due to hormonal changes in postmenopausal women [2,5,10]. The disease leads to joint damage, functional impairment, psychological distress, and a substantial decline in quality of life, imposing a heavy economic burden on patients and healthcare systems [2,4,5]. Effective management strategies for RA include early diagnosis, goal-oriented therapy, and patient education [2,11,12]. The European Alliance of Associations for Rheumatology (EULAR) emphasizes the importance of achieving remission or low disease activity to improve patient quality of life [13]. Regular monitoring and follow-ups are crucial in this context.

Advancements in information technology have enabled the emergence of smart healthcare interventions, which offer innovative, personalized, and remote approaches to disease management [14,15]. These interventions have demonstrated effectiveness in managing chronic diseases, including RA, by improving medication adherence and self-management skills while reducing outpatient visits and healthcare costs [16–19]. Smart healthcare leverages information and communication technologies (ICTs) to deliver disease management support remotely. This encompasses a continuum of services, ranging from passive dissemination of educational information to active symptom monitoring, personalized self-management support, and remote professional guidance. Despite the diversity in their specific applications, these interventions share a common core: the use of digital tools to transcend the spatiotemporal limitations of traditional clinic-based care, thereby providing continuous support to patients. Previous meta-analyses on this topic are limited, with the most recent study dating back to 2021. These studies encountered methodological challenges, such as inconsistent measurement scales and a lack of reported adverse events [20,21]. Given the rapid advancements in technology and new research findings, this updated meta-analysis aims to provide a comprehensive evaluation of the effectiveness of smart healthcare interventions for patients with RA, thereby enhancing the robustness of existing evidence.

## Methods

### Protocol and registration

This study was conducted following the Cochrane Handbook for Systematic Reviews of Interventions and the PRISMA guidelines [22,23]. The protocol was pre-registered with the International Prospective Register of Systematic Reviews (PROSPERO), registration number CRD42024582224.

### Data sources and search strategy

We systematically searched PubMed, Embase, and the Cochrane Library from inception to August 14, 2024, for relevant RCTs without language restrictions. Our search utilized Medical Subject Headings (MeSH) and keywords, including “Telemedicine,” “digital health,” “mHealth,” “Arthritis, Rheumatoid,” and “Randomized Controlled Trials.” Additionally, we reviewed reference lists of pertinent systematic reviews to enhance our search comprehensiveness. The details of the search strategy were shown in S1 File.

### Study selection and eligibility criteria

Eligibility criteria were defined based on the population, intervention, control, outcome, and study design. Two investigators independently assessed study eligibility after removing duplicates, the consistency of the reviews was quantified using Cohen’s Kappa coefficient (κ), and any discrepancies were resolved through discussion with a third reviewer (XL Li). Duplicates were removed prior to screening using the deduplication feature of NoteExpress (Version 4.0.0.9855). We included RCTs focused on smart health interventions for adult rheumatoid arthritis. We incorporated studies that defined RA through both established classification criteria (such as the 1987 American College of Rheumatology (ACR) criteria or the 2010 ACR/EULAR criteria) and those based on a physician’s clinical diagnosis. However, due to the lack of a uniform diagnostic standard across the literature, it was not feasible to apply a single, unified RA case definition across all studies. Eligible interventions encompassed all types of smart healthcare interventions, including those enabling symptom tracking and self-management support via health applications or online platforms, as well as telemedicine and teleconsultation services. Studies that did not report outcome data specifically attributable to these smart healthcare components were excluded. Common functions of the included interventions consisted of: automated symptom tracking (e.g., monitoring disease activity indicators such as DAS28 scores); regular remote follow-up (e.g., scheduled consultations via video or messaging); and self-management support (e.g., personalized medication reminders and exercise guidance).

Detailed characteristics of the smart healthcare interventions for RA are summarized in S2 File. The complete inclusion and exclusion criteria are provided in S3 File.

### Data extraction and outcome measures

Data extraction was performed independently by two authors, capturing details such as authorship, publication year, study size, duration, country, design, blinding, and participant demographics. We also recorded intervention types, comparison groups, and outcomes, including primary and secondary endpoints like disease activity, self-efficacy, quality of life, and adverse events. For incomplete data, authors were contacted for clarification.

The primary endpoint was the mean difference in disease activity, while secondary endpoints included self-management and functional changes.

### Quality assessment

The Cochrane Collaboration’s tool for assessing risk of bias was utilized [24]. Two independent reviewers (Y Yu and MJ Wang.) evaluated each study across the seven bias domains. Each item was classified as “low,” “unclear,” or “high” risk of bias. Any disagreements in assessments were resolved through discussion until consensus was achieved. When consensus could not be reached, a third senior reviewer (XL Li) was consulted to make the final determination.

### Statistical analysis

Statistical analyses were conducted using Stata software (version 14) with meta-analyses performed via a random-effects model to account for clinical heterogeneity. We calculated standardized mean differences (SMD) and 95% confidence intervals (CI) using the inverse variance method. Medians and interquartile ranges were converted to means and standard deviations [25,26]. Heterogeneity was assessed using the Cochrane Q test and quantified with the I^2^ statistic. A two-sided P-value <0.05 was considered statistically significant [27,28], and publication bias was not reported due to the limited number of included studies in each meta-analysis.

## Results

### Study identification

**Fig 1:**
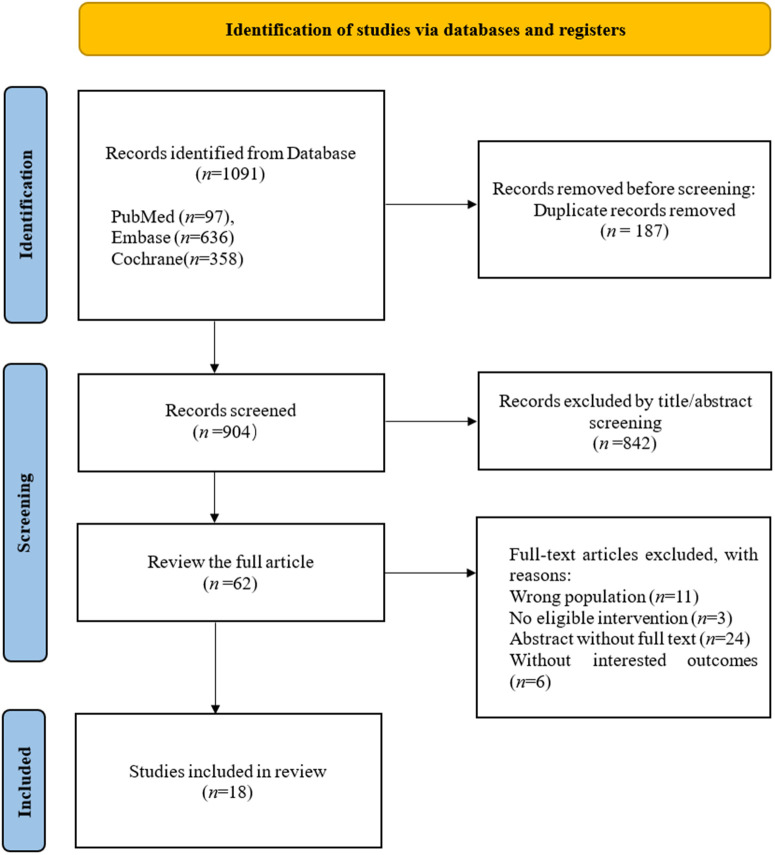
PRISMA flow diagram.

The initial search yielded 1,091 records. After removing 187 duplicates, we screened the titles and abstracts of the remaining 904 articles, excluding 842. We then evaluated the full text of 62 articles, resulting in the exclusion of 44, and ultimately included 18 RCTs in this meta-analysis [18,19,29–44]. The literature screening process is illustrated in Fig 1. The list of included and excluded articles along with the reasons can be found in S4 File.

### Study characteristics

The meta-analysis incorporated 18 RCTs published between 2015 and 2024. Of these, six studies were conducted in the Netherlands [31,36,37,39,41,42], three in Denmark [29,30,33], two in China [18,34], two in France [40], two in United States [35,38],and one each in Canada [31], Spain [44], Switzerland [43]. The majority of participants were women (median percentage across studies: 75.5%), with an average age of approximately 50–60 years. Thirteen studies reported disease activity [18,19,29–39], seven reported quality of life [29–31,37,40–42], two reported RA knowledge [29,43], two reported self-management [19,42], two reported functional level [32,36], and four reported adverse events [19,32,34,44]. Two studies addressed self-efficacy [29,42]. Key characteristics of the included RCTs are presented in Table 1.

**Table 1:** Characteristics of studies included in the meta-analysis.

Author	Country	Study period	Follow-up periods	Female/Male	Study Type	Control type	Age(Mean±SD)	Outcomes
Line R. Knudsen 2024	Denmark	2021–2023	12 months	I:54/35 C:56/35	Parallel, open-label, two-armed, randomized controlled trial	I: digital PE C: face-to-face PE	I: 59.0 (13.33) C: 59.0 (15.56)	Disease activity (DAS28 CDAI) Self-efficacy (RASE) Quality of life (EQ-5D) RA Knowledge (PKQ-RA)
Chun Li, 2023	China	November 1, 2018–May 2022	6 months	I: 903/196 C: 909/189	Multicenter, open-label, randomized clinical trial	I: smart system of disease management C: conventional care	I: 50.7 (12.4) C: 50.2 (12.5)	Disease activity (DAS28-CRP CDAI) Adverse Effects
Linda C Li 2023	Canada	2019–2022	26 weeks	I: 60/5 C: 60/6	Randomized controlled trial with a delayed-control design	I: a self-monitoring app, and PT counselling phone calls C: Delayed Group	I: 54.8 (13.1) C: 56.9 (13.2)	Disease activity (RADAI) Self-management (PAM) Adverse Effects
Bart P H Pouls 2022	Netherlands	August 2019–April 2021	3 months	I: 84/26 C: 78/33	Multicenter, randomized controlled trial	I: serious game C: conventional care	I: 61(12) C: 61(12)	Disease activity (RADAI) Functional level (HAQ)
Pablo Rodríguez Sánchez-Laulhé 2022	Spain	March 2020–February 2021	3 months	I: 9/5 C: 13/11	Single-blind, randomized controlled trial	I: CareHand app C: home exercise routine	I: 57.64 (7.25) C: 61.86 (10.76)	Adverse Effects
Bart Seppen 2022	Netherlands	May 2019–April 2020	12 months	I: 22/28 C: 21/32	Multicenter, randomized controlled trial	I: App-enabled patient proactive care C: usual care	I: 58(13) C: 57(11)	Disease activity (DAS28-ESR)
CV Skovsgaard 2023	Denmark	May 2014–July 2015	1 year	I: 89/47 C: 40/27	Randomized controlled trial	I: patient-reported outcome-based telehealth C: Conventional outpatient follow-up	I: 60.6 (12.2) C: 60.6(10.1)	Disease activity (DAS28) Quality of life (EQ-5D)
Bart F. Seppen 2023	Netherlands	May 2019–April 2021	12-month	I: 28/22 C: 32/21	Randomized controlled trial	I: patient-reported outcome-based telehealth C: usual care	I: 58 (13) C: 57 (11)	Disease activity (DAS28) Quality of life (EQ-5D)
Laurene Bernard 2022	France	August 16, 2016–July 29, 2019	6 months	I: 34/10 C: 33/12	Two-arm, randomized, pragmatic, controlled, prospective, monocentric, open-label clinical trial	I: monitored using a connected monitoring interface on a smartphone C: conventionally monitored	I: 55.9 (11.8) C: 55.5 (12.9)	Quality of life (EQ-5D)
Yvonne C. Lee 2021	USA	November 2016–May 2018	6 months	I: 73/18 C: 83/17	Randomized controlled trial	I: EPROs completed by an mobile app C: a care coordinator who provided data to patient’s rheumatologist.	/	Disease activity (CDAI)
Yuqing Song 2020	China	January 2015–December 2015	24 weeks	I: 30/11 C: 25/11	Unblinded, randomized controlled trial	I: educational sessions delivered through a telephone C: standard care	I: 57.05 (11.31) C: 53.22 (10.04)	Disease activity (DAS28)
Rixt Zuidema 2019	Netherlands	December 2014–June 2015	12 months	I: 51/27 C: 52/27	Multicenter exploratory randomized controlled trial	I: Web-based self-management program C: usual care	I: 61.0 (11.3) C: 62.9 (10.2)	Quality of life (Rand-36) Self-efficacy (RASE) Self-management (PAM)
Yves-Marie Pers 2021	French	April 2017–August 2019	6 months	I:33/11 C:34/10	Randomized, pragmatic, controlled, prospective, monocentric, open-label clinical trial	I: connected monitoring interface on smartphone C: conventional monitoring	/	Disease activity (DAS28) Functional level (HAQ) Adverse Effects
Maaike Ferwerda 2018	Netherlands	December 14, 2009–December 2013	1 year	I: 38/24 C: 47/24	Randomized controlled trial	I: internet-based cognitive behavioral therapy (ICBT) C: usual care	/	Quality of life (EQ-5D)
AnnetteThurah 2018(33)	Denmark	May 2014–July 2015	52 weeks	I: 63/25 C: 66/28	Pragmatic non-inferiority randomized controlled trial	I: PRO-based tele-health C: conventional out-patient	I: 61.6 (13.5) C: 60.7 (11.1)	Disease activity (DAS28)
Maaike Ferwerda 2017	Netherlands	December 14, 2009–December 2013	12 months	I: 38/24 C: 47/24	Parallel-group randomized controlled trial	I: internet-based tailored C: standard care	I: 55.45 (10.69) C: 57.14 (9.36)	Disease activity (RADAI) Quality of life (Rand-36)
Ahmed Allam 2015	Switzerland	November 1, 2012–July 31, 2013	4 months	I: 13/17 C: 13/12	Five-arm, parallel, randomized controlled trial	I: online social support and gamification C: no access to the website.	I: 55.10 (10.48) C: 69.33 (6.35)	RA Knowledge (PKQ-RA)
Brian J. Andonian 2024	USA	July 2021–February 2023	16 weeks	I: 7/3 C: 9/1	Randomized controlled trial	I: Supervised Weight loss and Exercise Training C: Counseling Health As Treatment	I: 67.7 (5.4) C: 65.6 (5.4)	Disease activity (DAS28-CRP DAS28-ESR)

Abbreviations: I = Intervention group; C = Control group; PE = Patient Education; RASE = Rheumatoid Arthritis Self-Efficacy; CDAI = Clinical Disease Activity Index; DAS28 = Disease Activity Score in 28 joints; PKQ-RA = Patient Knowledge Questionnaire (Rheumatoid Arthritis version); EQ-5D = EuroQoL 5-Dimension questionnaire; PAM = Patient Activation Measure; RADAI = Rheumatoid Arthritis Disease Activity Index; HAQ = Health Assessment Questionnaire; ESR = Erythrocyte Sedimentation Rate; CRP = C-Reactive Protein; PT = physiotherapist; ePROs = electronic patient reported outcomes

### Review consistency

The consistency of study selection was assessed using Cohen’s Kappa coefficient. Based on independent evaluations of 62 full-text articles, the κ value was 0.88 (95% CI 0.749–1.012), indicating almost perfect consistency. All discrepancies were resolved through discussion with a third reviewer.

### Risk of bias

**Fig 2:**
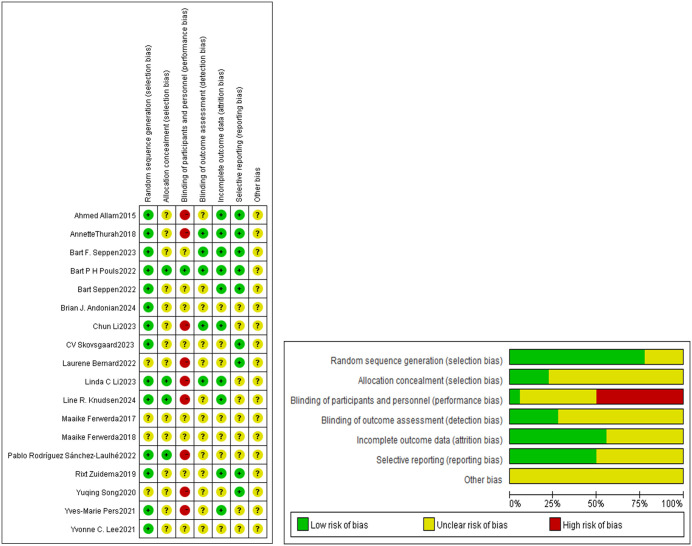
a. Risk of bias graph. b. Regarding disease activity, six articles reported on the Disease Activity Score (DAS28) [18,29,33]. The pooled analysis revealed a significant decrease in DAS28 scores (SMD with 95% CI: −0.22 [−0.38, −0.05], I^2^ = 28.8%, P = 0.011). Two articles reported on DAS28-CRP (SMD with 95% CI: −0.22 [−1.17, 0.40], I^2^ = 68.2%, P = 0.335) [34,38], while two articles reported DAS28-ESR (SMD with 95% CI: −0.06 [−0.70, 0.58], I^2^ = 49.1%, P = 0.863) [38,39]. Three studies reported on the Clinical Disease Activity Index (CDAI) (SMD with 95% CI: −0.12 [−0.30, 0.05], I^2^ = 51.6%, P = 0.169) [29,34,35], and three studies reported on the Rheumatoid Arthritis Disease Activity Index (RADAI) (SMD with 95% CI: −0.15 [−0.33, 0.04], I^2^ = 51.6%, P = 0.128) [19,36,37]. The results concerning disease activity are illustrated in Fig 3.

The risk-of-bias assessment is summarized in Fig 2. All studies utilized randomization; however, only 14 clearly described the random assignment process. Four studies implemented concealment methods, while the others did not specify any concealment procedures. Blinding of participants and personnel was identified as the domain with the highest risk of bias. Only one study maintained blinding for both parties, nine studies did not implement blinding at all, and the remaining studies did not report their blinding status. Five studies ensured blinding during outcome assessment, while others did not disclose whether blinding was applied. In terms of attrition bias, ten studies reported using intention-to-treat analyses or other statistical methods for data management, whereas eight studies remained unclear. For reporting bias, nine studies were deemed to have a low risk, while nine exhibited an unclear risk. Regarding other biases, all eighteen studies were classified as having an unclear risk. S5 File reports the risk of bias for each study.

**Fig 3:**
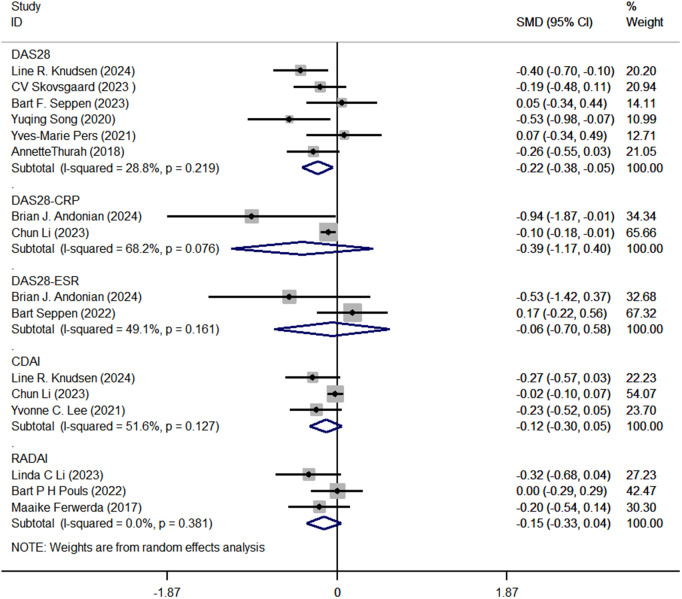
Forest plots of disease activity.

### Quality of life

**Fig 4:**
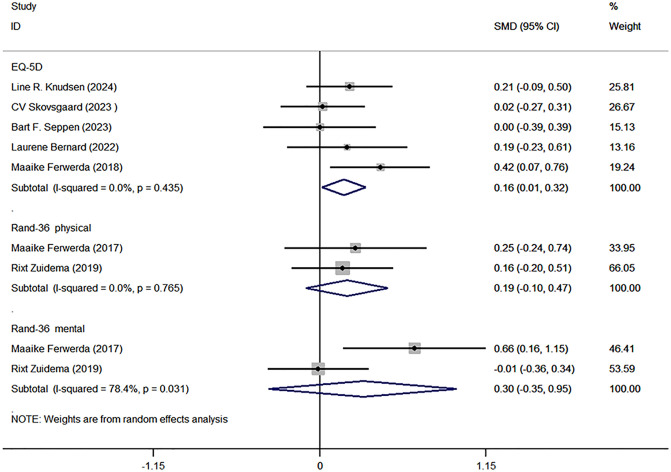
Forest plots of quality of life.

Quality of life was assessed using the EQ-5D and RAND-36 [45,46]. Five studies reported EQ-5D scores [29–31,40,41], confirming a significant difference between the intervention and control groups (SMD with 95% CI: 0.16 [0.01, 0.32], I^2^ = 0.0%, P = 0.033). Two studies analyzed RAND-36 [37,42], focusing on physical and mental health. The physical component showed no significant difference (SMD with 95% CI: 0.19 [−0.10, 0.47], I^2^ = 0.0%, P = 0.765), while the mental component demonstrated an unclear significance (SMD with 95% CI: 0.30 [−0.35, 0.95], I^2^ = 78.4%, P = 0.369) (Fig 4).

**Fig 5:**
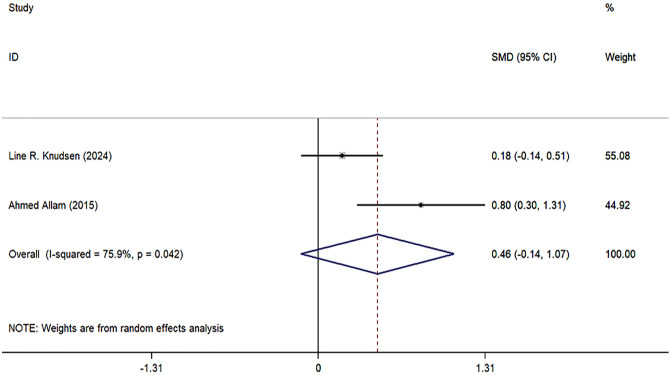
Forest plots of RA knowledge.

**Fig 6:**
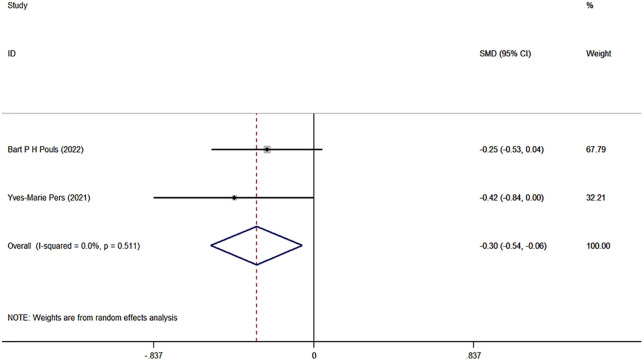
Forest plots of functional level.

**Fig 7:**
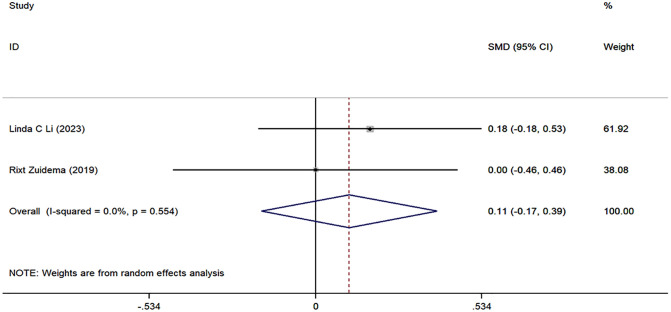
Forest plots of self-management.

**Fig 8:**
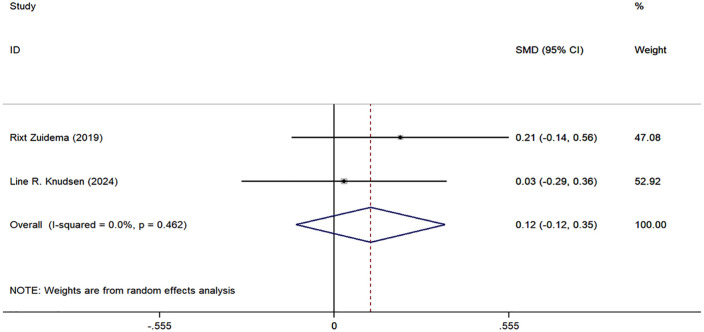
Forest plots of self-efficacy.

The meta-analysis on RA knowledge, functional level, self-management, and self-efficacy included only two studies. Although we report the combined effect sizes, caution should be exercised when interpreting these results due to the limited number of studies and potential methodological and clinical heterogeneity. The specific results are as follows: the combined effect size for RA knowledge was 0.46 (95% CI: −0.14, 1.07, I^2^ = 75.98%, P = 0.133) (Fig 5); the combined effect size for functional level was −0.30 (95% CI: [−0.54, −0.06], I^2^ = 0.0%, P = 0.014) (Fig 6); the combined effect size for self-management was 0.11 (95% CI: [−0.17, 0.39], P = 0.451) (Fig 7); and the combined effect size for self-efficacy was 0.12 (95% CI: [−0.12, 0.35], I^2^ = 0.0%, P = 0.340) (Fig 8).

### Adverse events

Adverse events were reported in four studies. Three studies noted adverse events unrelated to the intervention [19,34,44]. For example, one study reported muscle pain due to increased physical activity after initiating the intervention. Falls were also reported but were deemed unrelated to the intervention [19]. Another report mentioned that participants experienced disease-related pain episodes during the follow-up period [44]. Notably, one study recorded serious adverse events, including one death in the intervention group. The most prevalent serious adverse event in the control group was infection (n = 5), whereas only one case of infection was reported in the intervention group [32].

## Discussion

This systematic review and meta-analysis of 18 RCTs assessed the effectiveness of smart healthcare interventions for adults with RA, focusing on disease activity, self-efficacy, functional capacity, RA knowledge, self-management, and qualitative adverse events. Our findings reveal that smart healthcare interventions significantly reduce DAS28, enhance EQ-5D index values, and improve functional capacity, thereby elevating the quality of life for patients with RA. However, no statistically significant differences were observed in other outcome measures.

The methodological assessment revealed several issues requiring careful consideration. A primary concern was the inadequate reporting of allocation concealment procedures in multiple studies, resulting in uncertain risk of selection bias. Furthermore, nine studies demonstrated high risk of bias in blinding of participants and personnel – an inherent methodological challenge associated with the remote nature of digital interventions. Maintaining adequate blinding of participants, healthcare providers, and outcome assessors in digital health trials poses significant challenges, potentially compromising methodological rigor and leading to overestimation of treatment effects. Additionally, the limited number of available studies for certain outcome measures prevented us from conducting sensitivity analyses, thereby constraining the assessment of result robustness.

The analysis demonstrated substantial heterogeneity across multiple dimensions of the included interventions. The studies employed varied technological platforms (including mobile applications, teleconsultation systems, and web-based platforms), with differences in intervention types, implementation approaches, and follow-up durations (ranging from 3 to 12 months). While this diversity reflects the dynamic evolution of digital health technologies, it limited our ability to identify specific active components through subgroup analyses. Variations in outcome measurement instruments across studies (for instance, in RA knowledge assessment) further complicated cross-study comparisons [29,43]. The insufficient number of studies focusing on similar intervention components hindered meaningful subgroup analyses, highlighting the urgent need for standardized intervention frameworks and reporting guidelines in future trials.

An important finding relates to the demographic characteristics of the study populations. The average age of participants in most included studies exceeded 50 years, substantially limiting the generalizability of the findings. This age distribution may not adequately represent younger RA populations, who typically demonstrate different levels of digital literacy, technology usage patterns, communication preferences, and disease management needs. The effectiveness, engagement patterns, and implementation requirements of digital health interventions may differ significantly across age groups, indicating that current findings should be applied with particular caution to younger patient populations.

In contrast to previous meta-analyses that often combined different measurement instruments, our analytical approach involved separate evaluation of distinct measurement tools. We individually analyzed DAS28, DAS28-CRP, DAS28-ESR, CDAI, and RADAI for disease activity assessment, and independently evaluated EQ-5D and RAND-36 for quality of life measurement. This methodological refinement contributed to reduced clinical heterogeneity and enhanced the validity and reliability of our findings. However, the consistent underreporting of adverse events across the majority of studies represents a significant evidence gap that requires urgent attention in future digital health research.

## Conclusion

This systematic review indicates that digitally delivered healthcare interventions can improve key clinical outcomes in patients with RA, including disease activity, functional capacity, and quality of life. However, important limitations – including methodological weaknesses in the original studies, substantial clinical heterogeneity, and demographic considerations – necessitate cautious interpretation of the results and restrict their generalizability to younger populations. Future research should focus on establishing RA-specific standardized digital health frameworks; developing core outcome sets that incorporate both RA characteristics and digital health dimensions; conducting methodologically rigorous trials with appropriate blinding procedures; and performing stratified analyses to identify the most effective intervention components suitable for different age groups and patient characteristics. Particularly crucial is the implementation of large-scale randomized controlled trials that evaluate long-term efficacy, cost-effectiveness, and implementation strategies, while fully considering patient-specific factors such as digital literacy, technology access, and age-related characteristics.

## Supporting information

S1 File Search strategy. (DOCX)

S2 File Characteristics of Smart Healthcare Interventions. (DOCX)

S3 File Inclusion and Exclusion Criteria. (DOCX)

S4 File References of studies excluded based on full text. (XLS)

S5 File Risk of bias by RCT. (DOCX)

S6 File PRISMA Checklist. (DOCX)

S7 File PRISMA_2020_checklist. (DOCX)

## Abbreviations

RA: rheumatoid arthritis
CI: confidence interval
RCT: randomized controlled trial
SMD: standardized mean difference
PRISMA: Preferred Reporting Items for Systematic Reviews and Meta-Analyses
