# Supplementary material for: The effectiveness of smart healthcare for patients with rheumatoid arthritis: A systematic review and meta-analysis
Source: PLoS One. 2026 Jan 8;21(1):e0340074. doi: 10.1371/journal.pone.0340074 (PMC12782385; doi:10.1371/journal.pone.0340074)
Supplement: S1 File — (DOCX) [file pone.0340074.s001.docx]

**The retrieval strategies and retrieval results of each database are shown in Tables 1-2**.

Table 1: PubMed 2024.08.14

| No. | Content | Result |
| --- | --- | --- |
| #1 | Search: (((((("Telemedicine"[Mesh]) OR "Mobile Applications"[Mesh]) OR "Internet"[Mesh]) OR "Software"[Mesh]) OR "Digital Divide"[Mesh]) OR "Digital Health"[Mesh]) OR "Education, Distance"[Mesh] Sort by: Most Recent | 317,471 |
| #2 | Search: ((((((((((Mobile Health) OR (mHealth[Title/Abstract])) OR (health app[Title/Abstract])) OR (digital health[Title/Abstract])) OR (health monitoring app[Title/Abstract])) OR (telehealth[Title/Abstract])) OR (telediet*[Title/Abstract])) OR (Online Therapy[Title/Abstract])) OR (Digital Interventions[Title/Abstract])) OR (Computer Applications[Title/Abstract])) OR (Electronic Health Services[Title/Abstract]) Sort by: Most Recent | 128,358 |
| #3 | Search: ((((((("Telemedicine"[Mesh]) OR "Mobile Applications"[Mesh]) OR "Internet"[Mesh]) OR "Software"[Mesh]) OR "Digital Divide"[Mesh]) OR "Digital Health"[Mesh]) OR "Education, Distance"[Mesh]) OR (((((((((((Mobile Health) OR (mHealth[Title/Abstract])) OR (health app[Title/Abstract])) OR (digital health[Title/Abstract])) OR (health monitoring app[Title/Abstract])) OR (telehealth[Title/Abstract])) OR (telediet*[Title/Abstract])) OR (Online Therapy[Title/Abstract])) OR (Digital Interventions[Title/Abstract])) OR (Computer Applications[Title/Abstract])) OR (Electronic Health Services[Title/Abstract])) Sort by: Most Recent | 386,318 |
| #4 | Search: "Arthritis, Rheumatoid"[Mesh] Sort by: Most Recent | 129,640 |
| #5 | Search: (rheumatoid arthritis[Title/Abstract]) OR (reumat*[Title/Abstract]) Sort by: Most Recent | 127,618 |
| #6 | Search: ("Arthritis, Rheumatoid"[Mesh]) OR ((rheumatoid arthritis[Title/Abstract]) OR (reumat*[Title/Abstract])) Sort by: Most Recent | 172,924 |
| #7 | Search: (((((((("Telemedicine"[Mesh]) OR "Mobile Applications"[Mesh]) OR "Internet"[Mesh]) OR "Software"[Mesh]) OR "Digital Divide"[Mesh]) OR "Digital Health"[Mesh]) OR "Education, Distance"[Mesh]) OR (((((((((((Mobile Health) OR (mHealth[Title/Abstract])) OR (health app[Title/Abstract])) OR (digital health[Title/Abstract])) OR (health monitoring app[Title/Abstract])) OR (telehealth[Title/Abstract])) OR (telediet*[Title/Abstract])) OR (Online Therapy[Title/Abstract])) OR (Digital Interventions[Title/Abstract])) OR (Computer Applications[Title/Abstract])) OR (Electronic Health Services[Title/Abstract]))) AND (("Arthritis, Rheumatoid"[Mesh]) OR ((rheumatoid arthritis[Title/Abstract]) OR (reumat*[Title/Abstract]))) Sort by: Most Recent | 662 |
|  | Search: "Randomized Controlled Trials as Topic"[Mesh] Sort by: Most Recent | 176,884 |
|  | Search: (random*[Title/Abstract]) OR (RCT[Title/Abstract]) Sort by: Most Recent | 1,552,383 |
|  | Search: ("Randomized Controlled Trials as Topic"[Mesh]) OR ((random*[Title/Abstract]) OR (RCT[Title/Abstract])) Sort by: Most Recent | 1,612,048 |
|  | Search: (("Randomized Controlled Trials as Topic"[Mesh]) OR ((random*[Title/Abstract]) OR (RCT[Title/Abstract]))) AND ((((((((("Telemedicine"[Mesh]) OR "Mobile Applications"[Mesh]) OR "Internet"[Mesh]) OR "Software"[Mesh]) OR "Digital Divide"[Mesh]) OR "Digital Health"[Mesh]) OR "Education, Distance"[Mesh]) OR (((((((((((Mobile Health) OR (mHealth[Title/Abstract])) OR (health app[Title/Abstract])) OR (digital health[Title/Abstract])) OR (health monitoring app[Title/Abstract])) OR (telehealth[Title/Abstract])) OR (telediet*[Title/Abstract])) OR (Online Therapy[Title/Abstract])) OR (Digital Interventions[Title/Abstract])) OR (Computer Applications[Title/Abstract])) OR (Electronic Health Services[Title/Abstract]))) AND (("Arthritis, Rheumatoid"[Mesh]) OR ((rheumatoid arthritis[Title/Abstract]) OR (reumat*[Title/Abstract])))) Sort by: Most Recent | 97 |

Table 2 Embase 2024.08.14

| No. | Content | Result |
| --- | --- | --- |
| #1 | 'telemedicine'/exp OR 'mobile application'/exp OR 'internet'/exp OR 'software'/exp OR 'digital divide'/exp OR 'digital health'/exp OR 'distance learning'/exp | 862,172 |
| #2 | 'mobile health':ab,ti OR mhealth:ab,ti OR 'health app':ab,ti OR 'digital health':ab,ti OR 'health monitoring app':ab,ti OR telehealth:ab,ti OR telediet*:ab,ti OR 'online therapy':ab,ti OR 'digital interventions':ab,ti OR 'computer applications':ab,ti OR 'electronic health services':ab,ti | 38,041 |
| #3 | #1 OR #2 | 881,064 |
| #4 | 'rheumatoid arthritis'/exp | 267,108 |
| #5 | 'rheumatoid arthritis':ab,ti OR reumat*:ab,ti | 195,053 |
| #6 | #4 OR #5 | 292,080 |
| #7 | #3 AND #6 | 6100 |
| #8 | 'randomized controlled trial (topic)'/exp | 278,825 |
| #9 | random*:ab,ti OR rct:ab,ti | 2,109,660 |
| #10 | #8 OR #9 | 2,231,660 |
| #11 | #10 AND #7 | 636 |

Table 2 cochrane 2024.08.14

| #1 | MeSH descriptor: [Telemedicine] explode all trees | 4948 |
| --- | --- | --- |
| #2 | MeSH descriptor: [Mobile Applications] explode all trees | 2047 |
| #3 | MeSH descriptor: [Internet] explode all trees | 6624 |
| #4 | MeSH descriptor: [Software] explode all trees | 6216 |
| #5 | MeSH descriptor: [Digital Divide] explode all trees | 5 |
| #6 | MeSH descriptor: [Education, Distance] explode all trees | 313 |
| #7 | MeSH descriptor: [Digital Health] explode all trees | 15 |
| #8 | #1 OR #2 OR #3 OR #4 OR #5 OR #6 OR #7 | 16466 |
| #9 | (Mobile Health):ti,ab,kw | 17409 |
| #10 | (telehealth):ti,ab,kw OR (mHealth):ti,ab,kw OR (health app):ti,ab,kw OR (digital health):ti,ab,kw OR (health monitoring app):ti,ab,kw | 19396 |
| #11 | (telediet*):ti,ab,kw OR (Online Therapy):ti,ab,kw OR (Digital Interventions):ti,ab,kw OR (Computer Applications):ti,ab,kw OR (Electronic Health Services):ti,ab,kw | 31201 |
| #12 | #9 OR #10 OR #11 | 53762 |
| #13 | #8 OR #12 | 64417 |
| #14 | MeSH descriptor: [Arthritis, Rheumatoid] explode all trees | 8087 |
| #15 | (rheumatoid arthritis):ti,ab,kw OR (reumat*):ti,ab,kw | 19353 |
| #16 | #14 OR #15 | 19782 |
| #17 | #13 AND #16 | 461 |
| #18 | MeSH descriptor: [Randomized Controlled Trials as Topic] explode all trees | 55170 |
| #19 | (random*):ti,ab,kw OR (RCT):ti,ab,kw | 1364315 |
| #20 | #18 OR #19 | 1364430 |
| #21 | #17 AND #20 | 358 |
