# Supplementary material for: The effectiveness of smart healthcare for patients with rheumatoid arthritis: A systematic review and meta-analysis
Source: PLoS One. 2026 Jan 8;21(1):e0340074. doi: 10.1371/journal.pone.0340074 (PMC12782385; doi:10.1371/journal.pone.0340074)
Supplement: S2 File — (DOCX) [file pone.0340074.s002.docx]

**Table: Monitoring and Follow-up Characteristics of Smart Healthcare Interventions for Rheumatoid Arthritis (RA)**

| **No.** | **Author** | **Year** | **Intervention Type** | **Intervention Description** | **Monitoring Method** | **Follow-up Frequency** | **Technological Tools** |
| --- | --- | --- | --- | --- | --- | --- | --- |
| 1 | Line R. Knudsen | 2024 | Digital Patient Education | A digital patient education program with three modules (disease knowledge, medication management, symptom management) delivered via animations, videos, and interactive tests. | Electronic questionnaires, medical records, learning management system data | Baseline, 1, 3, 6, 12 months | Digital education platform, REDCap |
| 2 | Chun Li | 2023 | Mobile App Monitoring | Patients self-assess monthly via the SSDM app and upload data (DAS28-CRP, lab results, medication use, side effects); doctors monitor and adjust treatment online. | In-app data synchronization, lab reports, electronic medical records | Monthly, follow-ups at 6 and 12 months | SSDM smartphone app, Optical Character Recognition technology |
| 3 | Linda C. Li | 2023 | Wearable Device & App | Using Fitbit Inspire and OPERAS app to monitor physical activity, disease activity, and symptoms; combined with phone consultations from a physical therapist and action plan development. | Wearable device data, app records, phone consultation records | Twice a week (active phase) to biweekly | Fitbit Inspire, OPERAS app |
| 4 | Bart P H Pouls | 2022 | Gamification | Serious games (puzzles and behavioral tasks) combined with medication adherence education, with daily push reminders. | Game usage logs, electronic questionnaires, pill count | Baseline, 1, 3 months | Custom serious gaming app, CastorEDC data management software |
| 5 | Pablo Rodríguez Sánchez-Laulhé | 2022 | App-Based Exercise Program | The CareHand app provides hand rehabilitation training videos, pain recording, automatic adjustment of exercise intensity, along with diet and joint protection advice. | In-app pain recording, exercise completion tracking | Biweekly pain recording, weekly questionnaires | CareHand smartphone app (Android/iOS) |
| 6 | Bart Seppen | 2022 | Self-Monitoring App | Patients fill out the RAPID3 questionnaire weekly via the app, with an algorithm detecting disease fluctuations and triggering alerts; doctors can view data in real-time. | Electronic questionnaires, medical record synchronization | Weekly, 12-month follow-up | Custom smartphone app, EMR synchronization system |
| 7 | CV Skovsgaard | 2023 | Telehealth PRO System | Using the AmbuFlex system to complete PRO questionnaires (Flare-RA) every four months; doctors decide whether an in-person visit is needed based on the results. | Electronic questionnaires, lab data | Every four months | AmbuFlex remote PRO system |
| 8 | Bart F. Seppen | 2023 | Self-monitoring with PIC | Patients use an app for disease activity self-monitoring (RAPID3 score), receive lab results, and track health status. The app includes an alert system based on RAPID3 to notify patients of potential disease exacerbation. | Weekly 5-minute electronic patient-reported outcomes (RAPID3) | 12-month follow-up, patients can make extra appointments as needed | Mobile app (self-monitoring and health tracking), RAPID3 score, disease exacerbation alert algorithm, lab results integration |
| 9 | Laurene Bernard | 2022 | Connected Monitoring App | Using the SATIE-PR app, patients fill out RAPID3 and auto-DAS28 questionnaires weekly, combined with grip strength testing; clinical case managers coordinate alert responses. | In-app data, phone consultations, in-person visit records | Weekly, 6-month follow-up | SATIE-PR smartphone app, grip strength meter |
| 10 | Yvonne C. Lee | 2021 | PRO Monitoring App | Patients fill out RADAI-5 and PROMIS questionnaires daily via the app, with data synced in real-time to the doctor’s interface; care coordinators handle alerts. | Real-time data synchronization, phone follow-ups | Daily records, 6-month follow-up | Custom mobile app (Android/iOS) |
| 11 | Yuqing Song | 2020 | Telehealth Education | A phone-based educational intervention based on the health belief model (4 sessions), focusing on improving medication adherence and disease management skills. | Phone consultation records, electronic questionnaires, lab tests | Baseline, 12 weeks, 24 weeks | Phone follow-up system |
| 12 | Rixt Zuidema | 2019 | Web-Based Self-Management | An online self-management program with 9 modules (fatigue management, pain coping, etc.), with email reminders and nurse support. | Online questionnaires, usage logs | Baseline, 6, 12 months | Custom website platform |
| 13 | Yves-Marie Pers | 2021 | Connected Monitoring Interface | Using the SATIE-PR app combined with grip strength testing and PRO questionnaires; clinical case managers coordinate alert responses. | In-app data, in-person visit records | Weekly, 6-month follow-up | SATIE-PR app, grip strength meter |
| 14 | Maaike Ferwerda | 2018 | Internet-Based CBT | Personalized online cognitive behavioral therapy with four core modules (pain, fatigue, negative emotions, social function), combined with therapist-guided asynchronous communication. | Online questionnaires, therapist communication records | Baseline, 6, 9, 12 months | Custom online therapy platform |
| 15 | Annette Thurah | 2018 | Telehealth PRO System | Using Flare-RA questionnaires and CRP testing for remote disease activity monitoring; doctors decide follow-up based on the results. | Electronic questionnaires, lab data | Every four months | AmbuFlex system |
| 16 | Maaike Ferwerda | 2017 | Internet Cognitive Behavioral Therapy (iCBT) | Personalized treatment goal setting (in-person assessment), 4 core modules (pain/fatigue/negative emotions/social function), modular CBT strategies (cognitive restructuring, problem-solving, activity regulation), therapist-guided asynchronous online communication (weekly/bi-weekly). | Electronic task completion monitoring, paper questionnaire follow-ups (BDI/IRGL/CIS, etc.), patient self-assessment of therapy adherence | Follow-up after treatment (3/6/9/12 months) | Dedicated internet therapy platform (PC-based) |
| 17 | Ahmed Allam | 2015 | Web-Based Social Support | Online intervention combining social support and gamification elements (forums, achievement system), with a control group receiving only information. | Online interaction logs, questionnaires | Baseline, 2, 4 months | Custom website (including forum and gamification features) |
| 18 | Brian J. Andonian | 2024 | Remote Weight & Exercise Program | Remote supervised weight loss (MyFitnessPal records), aerobic/resistance training (custom video courses), combined with Garmin device to monitor steps and heart rate. | Electronic diet records, wearable device data, video course participation | Weekly, 16-week follow-up | MyFitnessPal, Garmin device, YouTube custom courses |
