# Supplementary material for: The effectiveness of smart healthcare for patients with rheumatoid arthritis: A systematic review and meta-analysis
Source: PLoS One. 2026 Jan 8;21(1):e0340074. doi: 10.1371/journal.pone.0340074 (PMC12782385; doi:10.1371/journal.pone.0340074)
Supplement: S3 File — (DOCX) [file pone.0340074.s003.docx]

Table: Inclusion and Exclusion Criteria

|  | Inclusion criteria | Exclusion criteria |
| --- | --- | --- |
| Population | Adults with rheumatoid arthritis | Children, adolescents, or multiple types of arthritis without providing independent data for patients  with RA |
| Intervention | Any type of smart health intervention including, but not restricted to, health apps, text messaging, computer, mobile phone, telephone, and online website interventions | Other behavior change techniques that are not based on smart health interventions  No separate data were provided for smart health interventions |
| Comparator | Routine care, waitlist control, or active control of not participating in other smart health programs | Comparators that incorporated smart health as intervention |
| Outcomes | disease activity, self-efficacy, quality of life, RA knowledge, Self-management ability, level of function, and adverse events | Not reporting disease activity, self-efficacy, quality of life, RA knowledge, Self-management ability, level of function, and adverse events |
| Study design | Randomised controlled trial | Observational, cross-sectional, pre/post, case studies, case series, case-control, systematic reviews, meta-analyses |
| Language | English | Non-English publications |
