# Supplementary material for: The effectiveness of smart healthcare for patients with rheumatoid arthritis: A systematic review and meta-analysis
Source: PLoS One. 2026 Jan 8;21(1):e0340074. doi: 10.1371/journal.pone.0340074 (PMC12782385; doi:10.1371/journal.pone.0340074)
Supplement: S5 File — (DOCX) [file pone.0340074.s005.docx]

S5.Risk of bias by RCT

| **Number** | **Author** | **Year** | **Random sequence generation (selection bias)** | **Allocation concealment (selection bias)** | **Blinding ofparticipants and personnel (performance bias)** | **Blinding of outcome assessment (detection bias)** | **Incomplete outcome data (attrition bias)** | **Selective reporting (reporting bias)** | **Other bias** |
| --- | --- | --- | --- | --- | --- | --- | --- | --- | --- |
| 1 | Line R. Knudsen | 2024 | low | low | high | unclear | low | unclear | unclear |
| 2 | Chun Li | 2023 | low | unclear | high | low | low | unclear | unclear |
| 3 | [Linda C Li](https://pubmed.ncbi.nlm.nih.gov/?sort=date&size=200&term=Li+LC&cauthor_id=38152927) | 2023 | low | low | high | low | low | unclear | unclear |
| 4 | Bart P H Pouls | 2022 | low | low | low | low | low | low | unclear |
| 6 | [Pablo Rodríguez Sánchez-Laulhé](https://mhealth.jmir.org/search?term=Pablo Rodr%C3%ADguez S%C3%A1nchez-Laulh%C3%A9&type=author&precise=true" \o "https://mhealth.jmir.org/search?term=Pablo Rodr%C3%ADguez S%C3%A1nchez-Laulh%C3%A9&type=author&precise=true) | 2022 | low | low | high | unclear | unclear | unclear | unclear |
| 5 | Bart Seppen | 2022 | low | unclear | unclear | unclear | low | low | unclear |
| 6 | CV Skovsgaard | 2023 | low | unclear | unclear | unclear | unclear | low | unclear |
| 7 | Bart F. Seppen | 2023 | low | unclear | unclear | low | low | low | unclear |
| 8 | Laurene Bernard | 2022 | unclear | unclear | high | unclear | unclear | low | unclear |
| 9 | Yvonne C. Lee | 2021 | low | unclear | unclear | unclear | unclear | unclear | unclear |
| 10 | Yuqing Song | 2020 | unclear | unclear | high | unclear | unclear | low | unclear |
| 11 | Rixt Zuidema | 2019 | low | unclear | unclear | unclear | low | low | unclear |
| 12 | Yves-Marie Pers | 2021 | low | unclear | high | unclear | low | unclear | unclear |
| 13 | [Maaike Ferwerda](https://www.jmir.org/search?term=Maaike Ferwerda&type=author&precise=true" \o "https://www.jmir.org/search?term=Maaike Ferwerda&type=author&precise=true) | 2018 | unclear | unclear | unclear | unclear | unclear | unclear | unclear |
| 15 | AnnetteThurah | 2018 | low | unclear | high | low | low | low | unclear |
| 16 | Maaike Ferwerda | 2017 | unclear | unclear | unclear | unclear | unclear | unclear | unclear |
| 17 | Ahmed Allam | 2015 | low | unclear | high | unclear | low | low | unclear |
| 18 | [Brian J. Andonian](https://acrjournals.onlinelibrary.wiley.com/authored-by/Andonian/Brian+J.) | 2024 | low | unclear | unclear | unclear | unclear | unclear | unclear |
