# Supplementary material for: The effectiveness of smart healthcare for patients with rheumatoid arthritis: A systematic review and meta-analysis
Source: PLoS One. 2026 Jan 8;21(1):e0340074. doi: 10.1371/journal.pone.0340074 (PMC12782385; doi:10.1371/journal.pone.0340074)
Supplement: S6 File — (DOCX) [file pone.0340074.s006.docx]

| **Section/topic** | **#** | **Checklist item** | **Reported – manuscript line number indicated to find start of each topic** |
| --- | --- | --- | --- |
| **TITLE** | | |  |
| Title | 1 | Identify the report as a systematic review, meta-analysis, or both. | Line 5 |
| **ABSTRACT** | | |  |
| Structured summary | 1 | Provide a structured summary including, as applicable: background; objectives; data sources; study eligibility criteria, participants, and interventions; study appraisal and synthesis methods; results; limitations; conclusions and implications of key findings; systematic review registration number. | Line 26 |
| **INTRODUCTION** | | |  |
| Rationale | 2 | Describe the rationale for the review in the context of what is already known. | Line 104 |
| Objectives | 3 | Provide an explicit statement of questions being addressed with reference to participants, interventions, comparisons, outcomes, and study design (PICOS). | Line 114 |
| **METHODS** | | |  |
| Protocol and registration | 4 | Indicate if a review protocol exists, if and where it can be accessed (e.g., Web address), and, if available, provide registration information including registration number. | Line 128 |
| Eligibility criteria | 4 | Specify study characteristics (e.g., PICOS, length of follow-up) and report characteristics (e.g., years considered, language, publication status) used as criteria for eligibility, giving rationale. | Line 132 |
| Information sources | 5 | Describe all information sources (e.g., databases with dates of coverage, contact with study authors to identify additional studies) in the search and date last searched. | Line 141 |
| Search | 4 | Present full electronic search strategy for at least one database, including any limits used, such that it could be repeated. | Line 149 |
| Study selection | 4 | State the process for selecting studies (i.e., screening, eligibility, included in systematic review, and, if applicable, included in the meta-analysis). | Line 183 |
| Data collection process | 5 | Describe method of data extraction from reports (e.g., piloted forms, independently, in duplicate) and any processes for obtaining and confirming data from investigators. | Line 192 |
| Data items | 5 | List and define all variables for which data were sought (e.g., PICOS, funding sources) and any assumptions and simplifications made. | Line 202 |
| Risk of bias in individual studies | 5 | Describe methods used for assessing risk of bias of individual studies (including specification of whether this was done at the study or outcome level), and how this information is to be used in any data synthesis. | Line 209 |
| Summary measures | 5 | State the principal summary measures (e.g., risk ratio, difference in means). | Line 225 |
| Synthesis of results | 5 | Describe the methods of handling data and combining results of studies, if done, including measures of consistency (e.g., I^2^) for each meta-analysis. | Line 236 |

Page 1 of 2

| **Section/topic** | **#** | **Checklist item** | **Reported on page #** |
| --- | --- | --- | --- |
| Risk of bias across studies | 5 | Specify any assessment of risk of bias that may affect the cumulative evidence (e.g., publication bias, selective reporting within studies). | Line 206 we indicated we searched for bias corrected data in the meta-analyses to make corrections/analyses in our results. Line 229 we indicated our check for higher and lower quality meta-analyses. |
| Additional analyses | 5 | Describe methods of additional analyses (e.g., sensitivity or subgroup analyses, meta-regression), if done, indicating which were pre-specified. | Line 241 we indicated our additional look at intervention vs. correlation based meta-analyses. |
| **RESULTS** | | |  |
| Study selection | 7 | Give numbers of studies screened, assessed for eligibility, and included in the review, with reasons for exclusions at each stage, ideally with a flow diagram. | Fig 2 is our flow diagram |
| Study characteristics | 8 | For each study, present characteristics for which data were extracted (e.g., study size, PICOS, follow-up period) and provide the citations. | Line 245; Table 1 is a comprehensive concerning the reviewed meta-analyses. |
| Risk of bias within studies | 13 | Present data on risk of bias of each study and, if available, any outcome level assessment (see item 12). | Line 288 |
| Results of individual studies |  | For all outcomes considered (benefits or harms), present, for each study: (a) simple summary data for each intervention group (b) effect estimates and confidence intervals, ideally with a forest plot. | Our goal was not a full meta-analysis of the meta-analyses. We provided Table 2 with the extracted effect size data and then Fig 2 with a visual of all effect size data. |
| Synthesis of results | 14 | Present results of each meta-analysis done, including confidence intervals and measures of consistency. | Appropriate summary data provided in Line 282 and Table 3 |
| Risk of bias across studies | 15 | Present results of any assessment of risk of bias across studies (see Item 15). | Line 278 |
| Additional analysis | 16 | Give results of additional analyses, if done (e.g., sensitivity or subgroup analyses, meta-regression [see Item 16]). | Line 303 and Table 3 |
| **DISCUSSION** | | |  |
| Summary of evidence | 20 | Summarize the main findings including the strength of evidence for each main outcome; consider their relevance to key groups (e.g., healthcare providers, users, and policy makers). | Line 318-358; Then recommendations beginning on Line 359. |
| Limitations | 21 | Discuss limitations at study and outcome level (e.g., risk of bias), and at review-level (e.g., incomplete retrieval of identified research, reporting bias). | Line 450 |
| Conclusions | 22 | Provide a general interpretation of the results in the context of other evidence, and implications for future research. | Line 465 |
| **FUNDING** | | |  |
| Funding | 23 | Describe sources of funding for the systematic review and other support (e.g., supply of data); role of funders for the systematic review. | We told you this in the submission upload process and cover letter. |

*From:*  Moher D, Liberati A, Tetzlaff J, Altman DG, The PRISMA Group (2009). Preferred Reporting Items for Systematic Reviews and Meta-Analyses: The PRISMA Statement. PLoS Med 6(7): e1000097. doi:10.1371/journal.pmed1000097

For more information, visit: **www.prisma-statement.org**.
